# Cognitive Development and Cannabis Use in Adolescents

**DOI:** 10.3390/bs11030037

**Published:** 2021-03-17

**Authors:** Alessandro Frolli, Maria Carla Ricci, Antonella Cavallaro, Agnese Lombardi, Antonia Bosco, Francesca Di Carmine, Francesca Felicia Operto, Luisa Franzese

**Affiliations:** 1 Disability Research Centre, University of International Studies of Rome, 00147 Rome, Italy; m.ricci@unint.eu (M.C.R.); f.dicarmine@unint.eu (F.D.C.); 2 FINDS—Italian Neuroscience and Developmental Disorders Foundation, 81040 Caserta, Italy; a-cavallaro@live.it (A.C.); lombardiagnese@gmail.com (A.L.); ant.bosco@hotmail.it (A.B.); 3 Department of Child Neuropsychiatry, ASL (Local Health Company) of Salerno, 84084 Salerno, Italy; opertofrancesca@gmail.com; 4 School, Regional School Office, 80136 Napoli, Italy; luisa.franzese@istruzione.it

**Keywords:** working memory, executive functions, adolescents, cannabis, cognition

## Abstract

Heavy exposure to cannabis during adolescence can cause significant neurocognitive changes. It can alter emotional responsiveness and social behavior, and cause impairments in sustained attention, learning, working memory (WM), cognitive flexibility, and the speed of information processing. It also has a significant impact on executive functions. In this study we investigated how global cognitive functions can be affected by the frequency of cannabinoid consumption in different categories of consumers (chronic, occasional, and non-users), through the evaluation of executive functions. Statistical analysis showed a significant decrease in performance in working memory tasks and processing speed by subjects using cannabis chronically (group 1) as compared to non-consumers (group 3), and occasional consumers (group 2). Future studies could verify the extent of neurocognitive alterations through re-evaluations with controlled follow-up and the addition of neuro-functional data.

## 1. Introduction

The use of narcotic substances often begins during adolescence, a period characterized by multiple neurocognitive changes and a reactivation in neurobiological development. In fact, from the age of 12 a new process of cerebral maturation leads to a global remodeling of the brain through phases of proliferation, migration, differentiation, synaptogenesis, and pruning [1,2,3,4]. The maturation of functional and cognitive processes involving the frontal and prefrontal cortex begins during adolescence. Final maturity with respect to higher cognitive functions is thus reached in that specific period [5]. Brain areas involved in this process are activated by new and exciting stimuli, as well as risky situations such as the abuse of alcohol, nicotine, and other substances that activate a complex, phylogenetically ancient brain circuit that plays a critical role in the search for the natural gratification essential for survival. This circuit is composed of the nucleus accumbens and the dopaminergic neurotransmission system, which is involved in craving phenomena [6]. It is important to note that the response of these areas to gratifying stimuli occurs with a greater intensity in adolescents, as they show reduced inhibitory control processes and a transient alteration of self-seeking functions (self-regulation). The immaturity of inhibitory control and self-regulation mechanisms, combined with hypersensitivity to gratification, explains the typical adolescent behavioral profile and the increased vulnerability that leads to the use of cannabis [7,8,9]. Furthermore, depending on additional variables (individual differences in the development process, different social contexts, and parental/educational influences), this experimentation can lead to drug addiction, which is caused by the chronic use of cannabis. The data available to date indicate that chronic cannabis use in adolescence can permanently modify some neuronal circuits in specific brain areas, and that such modifications could increase the likelihood of developing psychiatric disorders in adulthood [10,11]. Studies on the effects of cannabis use on cognitive functions highlight the appearance of deficits in sustained attention [12,13], in processing speed [14,15,16], in inhibitory control [15], in working memory [17,18], and in cognitive flexibility and the speed of information processing [19,20,21]. 

The evidence also suggests that individuals who start using cannabis at an early age may be more vulnerable to long-lasting neuropsychological deficits than those who start using it later [22]. Some studies have also shown a greater severity of neuropsychological deficits in chronic cannabis users [23,24]. In the definition of chronic use, most studies identify the temporal extension (duration) of use as a parameter and not the frequency. These studies evaluate cognitive deterioration through neurofunctional investigations [19,25,26,27,28,29,30] and through the administration of standardized neuropsychological tests [13,21,24,31,32,33,34]. 

In previous studies we validated our hypothesis related to the importance of both the temporal extension and the weekly frequency of use [1], and then investigated how the chronic use of cannabinoids caused serious impairments in cognitive function, in particular with respect to working memory and processing speed. However, the sample size was small. Thus, in the current study we decided to expand the sample number and further investigate the cognitive function of adolescent chronic consumers of cannabinoids, comparing it with that of a group of occasional consumers as well as with a control group of non-consumers. Specifically, we investigated how cannabis use (chronic, occasional, and non-use) can influence global cognitive functioning and executive functions, taking into consideration the weekly consumption frequency parameter and not just the temporal extension of use. We hypothesized that the group that used cannabis chronically (group 1) would show significant difficulties in the tasks relating to working memory (working memory index, WMI) and processing speed (processing speed index, PSI), with a consequent impact on the intellectual quotient (IQ), consistent with the findings highlighted by the literature [14].

## 2. Materials and Methods

### 2.1. Participants

For this study, 300 subjects between the ages of 15 and 16 were recruited from 10 secondary schools (high schools) in the province of Naples and from 10 secondary schools (high schools) in the province of Caserta. This study represents a collaboration between the Italian Foundation for Neuroscience and Developmental Disorders (FINDS) and the University of Salerno Centers for Child Neuropsychiatry, in partnership with the Regional School Office (USR) of Campania. This study was approved by the Academic Senate and the Ethics Committee of the University of International Studies of Rome (UNINT). The participation of the adolescents in the research was voluntary and was also approved by the parents. The data were collected anonymously, and at the end of the evaluations the schools granted training credit for research collaboration. Samples were pre-selected with a questionnaire aiming to record the children’s habits with respect to the use of cannabinoids. The questionnaire consisted of 6 questions. The first 5 questions involved a 5-item Likert scale for the expected response, and the sixth semi-open question concerned the quantification of cannabinoid use. The semi-open answer represented the core response to distinguish the subjects and assign them to the 3 groups. These groups were identified as: Group 1, comprising 100 subjects (Mean = 15.2; SD = 0.25—male 60/female 40) identified as chronic users of cannabis (at least 4 times a week for at least a year; group 2, which included 100 subjects (Mean = 15.5; SD = 0.14—male 70/female 30) who reported the occasional use of cannabis (about once every 2 weeks for at least 1 year); and group 3 (control), comprising 100 subjects (Mean = 15.3; SD = 0.12—male 65/female 35) who reported not using substances. The inclusion criterion determined that chronic use involved consumption 4 times weekly for at least a year, while occasional use was considered to be once every 2 weeks for at least a year. The control group did not use any substances. To eliminate the effects of the use of other substances, all those who regularly and frequently used other substances (tobacco or alcohol included) were excluded from the sample: the sample included only subjects who did not regularly and frequently use other drugs. All groups anonymously completed the WISC IV (Wechsler Intelligence Scale for Children -IV) [35] in the school environment by qualified psychologists belonging to the two reference clinics (FINDS and University of Salerno). The Corsi and Tower of London (ToL) subtests from the BVN (Neuropsychological assessment battery for the developmental age) 12–18 [36] were subsequently administered. The recruitment and evaluation procedures took 8 months. The three groups of subjects came from a homogeneous socio-cultural background. Moreover, in each group there were subjects from all schools in an equally distributed and randomized manner (Table 1).

### 2.2. Procedures and Tasks

The protocol used in this study was composed of a specifically constructed questionnaire which investigated the frequency of cannabinoid consumption and the following tests: the WISC IV, the BVN 12–18, and the MT Trials-Advanced 3 (Assessment test of reading and reading comprehension skills and math skills) [37]. The used tools all had a high reliability rate, and included the most recent Italian standardized versions.

Questionnaire: The questionnaire included 5 closed questions that investigated the relationship with and perception of cannabinoids, e.g., “Do you generally use cannabinoids in adolescence?” (question 2). Answers were given on a 5-point Likert scale ranging from one (never) to five (often). Finally, a sixth semi-open question was provided; this question was used to investigate weekly use. On the basis of this last question, the subjects were divided into 3 groups (chronic, occasional, and non-users).

WISC-IV: This is a clinical and diagnostic tool used to assess the intellectual abilities of children aged 6 to 16. It consists of 15 tests (10 main and 5 supplementary) divided into 4 indices. The 10 main tests are represented by: block design (BD), similarities (SI), digit span (DS), picture concepts (PCN), coding (CD), vocabulary (VC), letter–number sequencing (LN), matrix reasoning (MR), comprehension (CO), and symbols search (SS). They are divided into 4 indices: The perceptual reasoning index (PRI), which includes BD, PCN, and MR; the verbal comprehension index (VCI), which includes SI, VC, and CO; the working memory index (WMI) which includes DS and LN; and the processing speed index (PSI), which includes CD and SS.

BVN 12–18: This is a test battery for neuropsychological evaluation to identify single disorders in specific areas and to define a general profile of mnemonic, praxic, visuospatial, perceptive, attentive, linguistic, and executive skills, etc., that are useful for deeper study after an initial diagnostic assessment. In particular, the Tower of London (ToL) subtest evaluates higher executive functions such as planning and problem-solving skills. The Corsi subtest, on the other hand, allows us to evaluate the span of visuospatial memory, that is, the amount of visuospatial information that can be retained in short-term memory (MBT).

MT Trials-Advanced 3: These tests thoroughly assess reading, text comprehension skills, and math skills in adolescents. They include word and non-word reading tests, text comprehension tests, writing tests by dictation, and tests of arithmetic skills and facts.

#### 2.2.1. Procedures

All subjects included in the sample were administered the questionnaire described above to investigate perceptions, relationships, and use regarding cannabinoids. Following the analysis of the responses (in particular to the sixth question), we divided the sample into 3 groups: group 1, which reported the chronic use of cannabinoids (at least 4 times a week for at least a year); group 2, which reported occasional use (about once every 2 weeks for at least 1 year); and group 3, which reported not using cannabinoids. The third group was obtained from a randomization of those who did not use substances, as emerged from the questionnaire. All groups then completed the WISC-IV, using the standardized Italian version. The scores on the individual tasks were analyzed. In particular, the main indices were: VCI, PRI, WMI, PSI, and IQ, namely the perceptual reasoning index (PRI) including BD, PCN, and MR; the verbal comprehension index (VCI) including the SI, VC, and CO; the working memory index (WMI) including DS and LN; and the processing speed index (PSI) including the CD and SS. We hypothesized that the group that used cannabis chronically (group 1) would have significant difficulties in undertaking the tasks relation to working memory (WMI) and processing speed (PSI), with a consequent impact on the intellectual quotient (IQ), consistent with the findings highlighted by the literature (Fried et al., 2005). We then administered the subtests of the BVN 12–18 for an in-depth study of memory skills (visuospatial) (course test) and planning (subtest of the London Towers). Our hypothesis was that the group of subjects who used cannabis chronically (group 1) would have significantly reduced scores in tasks and planning (ToL) and visuospatial memory (Corsi) compared to the control group (group 3). In addition, to estimate the percentage of specific learning disabilities, we administered the MT Trials-Advanced 3: to the entire population.

#### 2.2.2. Methods

Data analyses were performed using SPSS 26.0 (IBM, 2019, New York City, NY) statistical survey software [38]. Significance was accepted at the 5% level (*p* < 0.05). We compared the weighted scores of the groups to the indices (VCI, PRI, WMI, PSI, IQ) that emerged from the WISC-IV and the scores that emerged from the ToL and Corsi sub-tests through the use of the analysis of variance test (ANOVA), a parametric test that allows for the comparison two or more data groups by comparing the variability within these groups with the variability between groups. The relationship between these variances follows the Fisher F distribution, which allows for an examination of the hypotheses regarding the significance of the difference between the variability due to treatment and the residual variability. In this study, we performed an ANOVA to compare the scores between the groups.

## 3. Results

The comparison between the three groups regarding the WISC-IV showed a significant effect of the group on the following indices: VCI (F (2, 299) = 9.968, *p* < 0.05), PRI (F (2, 299) = 12.588, *p* < 0.05), WMI (F (2, 299) = 314.113, *p* < 0.05), PSI (F (2, 299) = 47.539, *p* < 0.05), and IQ (F (2, 299) = 119.343, *p* < 0.05). The comparison between the three groups also revealed a significant effect on the ToL test (F (2, 299) = 29.007, *p* < 0.05) and on the Corsi test (F (2, 299) = 79.222, *p* < 0.05) (Table 2).

To understand the differences between the individual groups (and verify our hypothesis) we performed post hoc tests (Bonferroni). From this analysis, it emerged that the scores of group 1 differed significantly from those of group 3 for the following indices: VCI (F (2, 299) = −3.020, *p* < 0.05), PRI (F (2, 299) = −3.770, *p* < 0.05), WMI (F (2, 299) = −15.570, *p* < 0.05), PSI (F (2, 299) = −7.260, *p* < 0.05), and IQ (F (2, 299) = −13.980, *p* < 0.05), showing that chronic use of cannabinoids had a significant impact on cognitive abilities and visual-perceptive reasoning, and, to a greater extent, on IQ, working memory functions, and processing speed. Significant differences also emerged with regard to the ToL subtest (F (2, 299) = −1.150, *p* < 0.05) and the Corsi subtest (F (2, 299) = −1.550, *p* < 0.05). These results showed that chronic use of cannabinoids can impair planning and visuospatial memory skills as compared to non-consumers (group 3) (Table 3).

The comparison between group 1 and group 2 revealed differences in the following indices: VCI (F (2, 299) = −2.940, *p* < 0.05), PRI (F (2, 299) = −3.380, *p* < 0.05), WMI (F (2, 299) = −12.810, *p* < 0.05), PSI (F (2, 299) = −6.120, *p* < 0.05), and IQ (F (2, 299) = −11.340, *p* < 0.05), showing that chronic cannabis use had an impact on verbal comprehension and visual-perceptual reasoning, and to a greater extent on IQ, working memory functions, and processing speed. We then compared the performances of the ToL subtest (F (2, 299) = −0.940, *p* < 0.05) and Corsi (F (2, 299) = −1.270, *p* < 0.05), and significant differences emerged. These results show that chronic use of cannabinoids had an impact on planning skills and visuospatial memory skills when compared to those who used them less frequently (Table 4).

**Figure 1:**
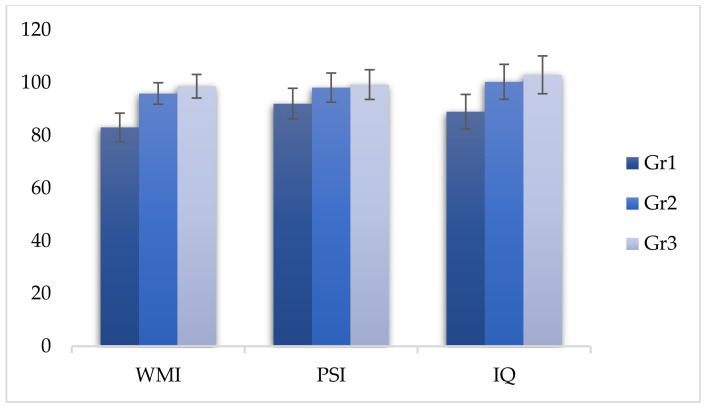
Comparison of WISC-IV (Wechsler Intelligence Scale for Children -IV) indices between the three groups.

**Figure 2:**
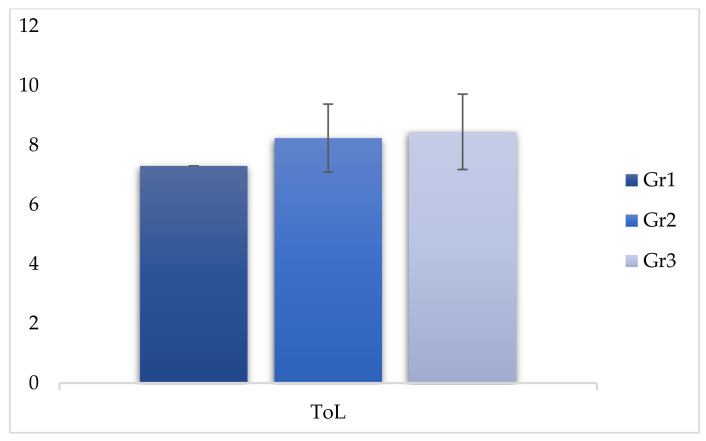
Comparison of ToL (Tower of London) between the three groups.

**Figure 3:**
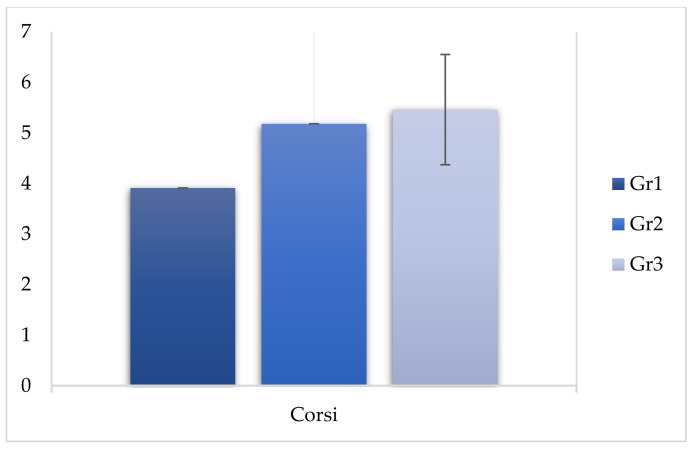
Comparison of Corsi between the three groups.

The comparison between the scores of group 2 and group 3 revealed significant differences in the WMI index (F (2, 299) = −2.760, *p* < 0.05), demonstrating that even infrequent use of cannabinoids affected the performance of working memory (Table 5 and Figure 1, Figure 2 and Figure 3).

**Figure 4:**
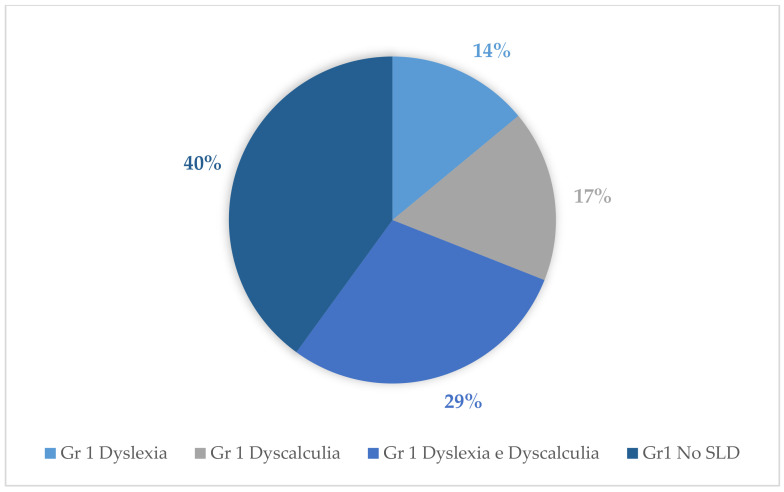
Percentages of SLD (specific learning disabilities) in group 1.

**Figure 5:**
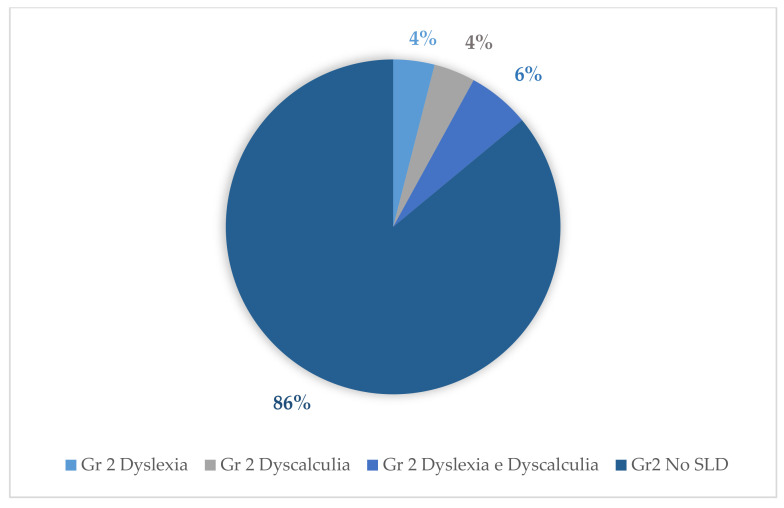
Percentages of SLD in group 2.

**Figure 6:**
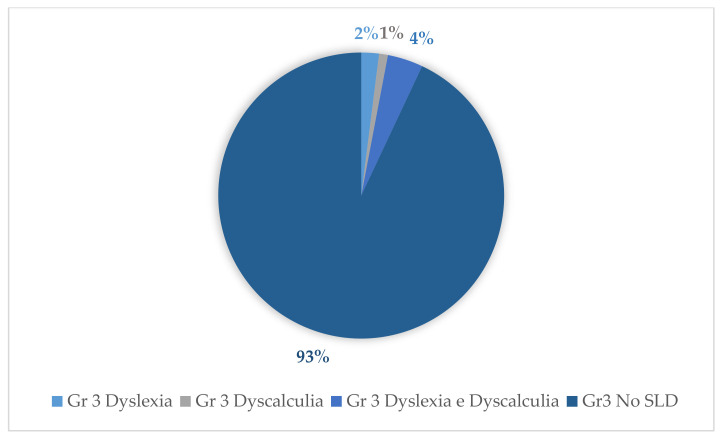
Percentages of SLD in group 3.

Tests for the evaluation of reading and mathematical skills of the assessed cases revealed the following percentages for the diagnosis of dyslexia and dyscalculia: With regard to group 1, 14% (14 subjects) were diagnosed with dyslexia and 17% (17 subjects) were diagnosed with dyscalculia. Of these, 29% (29 subjects) were diagnosed with both dyscalculia and dyslexia. With regard to group 3, 2% (2 subjects) were diagnosed with dyslexia and 1% (1 subject) was diagnosed with dyscalculia; of these, 4% (4 subjects) were diagnosed with both dyslexia and dyscalculia. Concerning group 2, the following percentages emerged: 4% (4 subjects) were diagnosed with dyslexia and 4% (4 subjects) were diagnosed with dyscalculia. Of these, 6% (6 subjects) were diagnosed with both dyslexia and dyscalculia (Figure 4, Figure 5 and Figure 6).

## 4. Discussion

Several studies have shown a high rate of cannabinoid consumption among adolescents [9,13], showing that with greater ease of access to drugs, precocious use increased. Persistent use of cannabis before the age of 18 can cause permanent cognitive damage to function in cognition, attention, and memory [13,17,18]. Furthermore, stopping the consumption does not seem to restore cognitive functions [11]. Age is considered to be a key variable. In several studies [39,40] it emerged that those who started abusing cannabinoid substances after the age of 18 did not show an equal decline in the cognitive functions. The reason may lie in the fact that before the age of 18 the brain is still in the phase of organization and restructuring (the pruning phase is still in progress), and therefore it appears to be more vulnerable to damage resulting from the use of drugs. In subjects who use cannabis chronically, the volume and shape of the hippocampus appears to be different with respect to the general population, and this gap seems to widen in relation to the length of the period of consumption of the substance [41,42]. Other studies have reported that cannabis use also influences cognitive functions. More specifically, they have revealed diminished attention, learning, working memory, and information processing speed [19,20,21]. Further studies have investigated the impact of early onset cannabis use on cognitive functioning and impairment of executive functions regardless of increased frequency and extent of use [13,21,32,33,34,43]. In a review, Solowij and Battisti [24] tried to clarify the extent to which cognitive impairment (in particular working memory) can persist beyond acute intoxication, but did not focus on the frequency of use. The novelty of our study is therefore the consideration of weekly attendance as a fundamental parameter to discriminate the results. The statistical analysis in our study showed that the group of subjects who used cannabis chronically (group 1, who used of cannabinoids four times a week for at least a year) showed significantly reduced performance in working memory tasks as compared to the group that did not take drugs (group 3). In addition, there was also a reduction in the performance of tasks relating to information processing speed and IQ, highlighting a significant difference with regard to the control group (group 3). Analyzing the scores of the group that instead used cannabis occasionally (group 2, which used cannabinoids once every two weeks for at least a year), a reduction in working memory emerged as compared to the control group (group 3), thus confirming the significant impact of frequency in the use of cannabinoid substances. The comparison between group 1 and group 2 also showed significantly diminished working memory, processing speed, and IQ in group 1 as compared to group 2. The results of this study highlight the fact that the use of substances in adolescence (an early and sensitive age due to the new brain maturation process) has a significant impact on neurocognitive alterations [44,45], particularly on cognitive processes such as working memory and processing speed and especially when use is chronic (highly frequent use of the substance). Therefore, the impact of cannabis use on cognitive function highlighted by previous studies [15,46] must be considered not only in terms of temporal extension (duration) but also of frequency. Furthermore, with regard to the neuropsychological study carried out on our sample, the statistical analysis showed that the group that chronically used cannabis (group 1) presented with a significant impairment in planning skills as compared to the control group (group 3), with an impact of the substance found also on some executive functions such as planning. Chronic cannabis use was found to have a significant impact on the functioning of higher cognitive processes such as working memory skills, processing speed, and planning skills, to a greater extent than the group which did not use cannabinoids (group 3). In addition, our analysis suggests that the group that chronically used cannabinoids (group 1) performed poorly in higher visual-spatial material processing skills as compared to the non-cannabinoid-using group (group 3). Data reinforcing the hypothesis of a significant impact between the frequency of cannabis use and specific alterations in working memory only emerged in the comparison between group 2 and group 3 in the WISC IV working memory tests. Some studies have already described the chronic effects of heavy cannabis use on the brain and how these could induce even more marked cognitive problems in adolescents [47].

In particular, in recent decades there has been a marked increase in the prevalence of cannabis strains that have a high percentage of the psychoactive constituent tetrahydrocannabinol (THC) [48], which exerts persistent effects on cognition and mental and brain health [49,50], while also generating greater addiction (Lupica et al., 2004). From a neurobiological point of view, several preclinical studies have also shown that THC is neurotoxic to brain areas rich in type-1 cannabinoid receptors, including the hippocampus [51,52,53,54,55], the amygdala [55], the striatum [56], and the prefrontal cortex (PFC) [56,57,58], as documented through the use of functional magnetic resonance imaging (MRI). Other studies have found associations between higher cannabis dosage and hippocampal alterations, and between earlier age of onset and PFC alterations [59]. Cannabis users showed neuroanatomical alterations in both regions. These regions concern reward [60], memory [61] and executive attention systems [62,63], and can mediate deficits that cannabis users show in these domains [60,61,62,63,64,65,66,67]. These neuroanatomical alterations could also be called into question in our study to explain the various highlighted deficits, including above all those of working memory.

A more recent study, conducted by Morin et al., [68], also found that cannabis use in adolescence was associated with generally lower performance in working memory (WMI), perceptual reasoning (PRI), and processing speed (PSI), although it did not discriminate frequency well. In fact, in our study we proposed weekly use as a fundamental parameter for discriminating outcomes. The results of this study, in accordance with the literature, make it clear that subjects who start chronic weekly consumption at an early age (especially in adolescence) are more vulnerable to cognitive deficits. In addition, some studies have found learning deficits [39,69]. Our study analyses have in fact highlighted the presence of a greater extent of specific learning disorders in the group of chronic users. With regard to group 1 in particular, 14% (14 subjects) were diagnosed with dyslexia and 17% (17 subjects) were diagnosed with dyscalculia. Of these, 29% (29 subjects) were diagnosed with both dyscalculia and dyslexia. These data are consistent with the findings of previous studies on the presence of ASD (Autism Spectrum Disorder) in adolescents who chronically used cannabinoids [39,69]. However, in our sample, information emerged specifically on subjects with a diagnosis of both dyslexia and dyscalculia. In contrast, for group 3 the following percentages emerged: 2% (2 subjects) were diagnosed with dyslexia and 1% (1 subject) was diagnosed with dyscalculia; of these, 4% (4 subjects) were diagnosed with both dyslexia and dyscalculia. Finally, with regard to group 2, the following percentages emerged: 4% (4 subjects) were diagnosed with dyslexia and 4% (4 subjects) were diagnosed with dyscalculia; of these, 6% (6 subjects) received diagnoses of both dyslexia and dyscalculia.

## 5. Conclusions and Limitations

Considering that the socio-cultural class of the selected adolescents was medium–high, the number of recruited subjects, and the strong significance of our data, our data suggest a significant impact between chronic use of cannabinoids and a reduction in the efficiency of working memory and processing speed. Longer follow-up and an experimental procedure could confirm the stability of the data and allow for the consideration of the deficits found as permanent. Furthermore, it would be useful, in future research, to create a T0, with assessments of the IQ and other indices of the WISC IV. This T0 should represent the pre-initiation phase with regard to cannabis use and could effectively establish the correlation between cognitive factors and cannabis use. In fact, in our study, as the IQ before starting cannabis use was unknown, it is not possible to state unequivocally that it was the chronic use of cannabis that lowered the cognitive indices. Specifically, pre-existing cognitive vulnerability could act as a predisposing factor for chronic cannabis addiction or use. Further studies could address the comparison between the effects in the population of adults and adolescents exposed to different gradients of frequency of use. The repercussions in terms of prevention and rehabilitation are also important. Another valuable consideration derived from this work is that, given their developmental stage and neurobiological predisposition, adolescents may be particularly vulnerable: thus, it is necessary to identify use of cannabis in a timely manner and seek effective treatments to discontinue the consumption of the substance [70]. Access to specific education and prevention programs should also be introduced in school settings.

## Figures and Tables

**Table 1:** Subdivision of the sample.

Group 1			Group 2			Group 3		
M_age_	SD	Gender	M_age_	SD	Gender	M_age_	SD	Gender
15.2	0.25	M/F 60/40	15.5	0.14	M/F 70/30	15.3	0.12	M/F 65/35

**Table 2:** Comparison of indices between group 1, group 2, and group 3. VCI: verbal comprehension index; PRI: perceptual reasoning index; WMI: working memory index; IQ: intellectual quotient; ToL: Tower of London.

Indices	Group1		Group2		Group3		F	*P*
	Means	SD	Means	SD	Means	SD		
**VCI**	98.76	4.57	101.70	6.30	101.78	5.33	9.968	<0.05 *
**PRI**	99.12	6.61	102.50	5.88	102.89	4.88	12.588	<0.05 *
**WMI**	83.02	5.41	95.83	4.08	98.59	4.46	314.113	<0.05 *
**PSI**	91.99	5.81	98.11	5.53	99.25	5.63	47.539	<0.05 *
**IQ**	88.94	6.57	100.28	6.64	102.92	7.16	119.343	<0.05 *
**ToL**	7.30	0.969	8.24	1.14	8.45	1.27	29.007	<0.05 *
**Corsi**	3.91	0.740	5.18	0.914	5.46	1.09	79.222	<0.05 *

* *p* < 0.05

**Table 3:** Post hoc comparisons between group 1 and 3.

Indices	Group 1		Group 3		Difference in Means	*p*
	Means	SD	Means	SD		
**VCI**	98.76	4.57	101.78	5.33	−3.020	<0.05 *
**PRI**	99.12	6.61	102.89	4.88	−3.770	<0.05 *
**WMI**	83.02	5.41	98.59	4.46	−15.570	<0.05 *
**PSI**	91.99	5.81	99.25	5.63	−7.260	<0.05 *
**IQ**	88.94	6.57	102.92	7.16	−13.980	<0.05 *
**ToL**	7.30	0.969	8.45	1.27	−1.150	<0.05 *
**CORSI**	3.91	0.740	5.46	1.09	−1.550	<0.05 *

* *p* < 0.05

**Table 4:** Post hoc comparisons between group 1 and 2.

Indices	Group 1		Group 2		Difference in Means	*P*
	Means	SD	Means	SD		
**VCI**	98.76	4.57	101.70	6.30	−2.940	<0.05 *
**PRI**	99.12	6.61	102.50	5.88	−3.380	<0.05 *
**WMI**	83.02	5.41	95.83	4.08	−12.810	<0.05 *
**PSI**	91.99	5.81	98.11	5.53	−6.120	<0.05 *
**IQ**	88.94	6.57	100.28	6.64	−11.340	<0.05 *
**ToL**	7.30	0.969	8.24	1.14	−0.940	<0.05 *
**CORSI**	3.91	0.740	5.18	0.914	−1.270	<0.05 *

* *p* < 0.05.

**Table 5:** Post hoc comparisons between group 2 and 3.

Indices	Group 2		Group 3		Difference in Means	*p*
	Means	SD	Means	SD		
**VCI**	101.70	6.30	101.78	5.33	−0.80	1.000
**PRI**	102.50	5.89	102.89	4.88	−0.390	1.000
**WMI**	95.83	4.08	98.59	4.46	−2.760	<0.05 *
**PSI**	98.11	5.53	99.25	5.63	−1.140	0.467
**IQ**	100.28	6.64	102.92	7.16	−2.640	0.019
**ToL**	8.24	1.14	8.45	1.27	−0.210	0.578
**CORSI**	5.18	0.914	5.46	1.09	−0.280	0.101

* *p* < 0.05.

## Data Availability

The data presented in this study are available on request from the corresponding author.
